# A crowdsourcing approach to collecting photo-based insect and plant observation records

**DOI:** 10.3897/BDJ.5.e21271

**Published:** 2017-11-06

**Authors:** Takeshi Osawa, Takehiko Yamanaka, Yukinobu Nakatani, Jun Nishihiro, Shiori Takahashi, Suzuki Mahoro, Hironobu Sasaki

**Affiliations:** 1 Institute for Agro-Environmental Sciences, NARO, Tsukuba, Japan; 2 Faculty of Science, Toho University, Funabashi, Japan; 3 Iwate Prefetural Museum, Morioka, Japan; 4 Sumisaka Junior High School, Sumisaka, Japan

**Keywords:** Citizen science, Mobile phone, Social networking system, Open data

## Abstract

**Background:**

Scientific field observation by members of the public is known as citizen science and has become popular all across the world. Citizen science is advantageous for collecting large amounts of scientific data and can be seen as a crowdsourcing approach to data collection. Information and communications technology is enhancing the availability of citizen science. Mobile devices, such as mobile phones, that have a digital camera with a global positioning system (GPS) are necessities for contemporary life and can be utilised as powerful observation tools in citizen science.

**New information:**

A web-based system has been developed as a data collection tool for citizen science. Participants submit an e-mail with a photo taken by their mobile phones. The photos contain location information, which can be easily and automatically embedded if the mobile phone is equipped with GPS. Collaboration has been undertaken with regional event managers, such as museum curators and held citizen science events in each region and for various target taxonomic groups. All photos were stored in the data server and the organisms were taxonomically identified by citizen scientists, regional managers and the authors. In total, 154 species and 843 data records were collected in this project conducted from 2011 to 2016.

## Introduction

A citizen scientist is a volunteer who collects or processes data as part of a scientific enquiry (Silvertown 2009). Today, these volunteers are active in several scientific areas, especially the environmental sciences (Dickinson et al. 2010, Dickinson et al. 2012) and many recent studies have benefitted from this approach (Osawa 2015, Miyazaki et al. 2016, Sullivan et al. 2009, Osawa 2013). One of the important research benefits provided by citizen science is the crowdsourcing of large data sets (Osawa 2015). In addition to its scientific merits, this approach has educational advantages because it gives members of the public opportunities to observe and interact with organisms in nature (Dickinson et al. 2010, Dickinson et al. 2012, Kobori et al. 2015, Miyazaki et al. 2014). Thus, the approach can provide substantial benefits for both researchers and citizens.

Recent advances in information and communications technology have made citizen science more user-friendly and accessible (Kobori et al. 2015, Silvertown 2009). Mobile devices, in particular, have become key tools for citizen science activities in recent years (Silvertown 2009). Most mobile phones have a digital camera with a global positioning system (GPS) that allows people to record species occurrence data with high precision, because the data contain not only the location details, date and time but also the evidence (i.e. photo) of the targets (Osawa et al. 2013). As mobile phones have become a necessity for contemporary life, citizen science projects using mobile phones are expected to attract more volunteers than ever before (Osawa et al. 2013).

A web-based system has been developed as a data collection tool for citizen science. When a participant submits an e-mail with a photo that has embedded GPS information, the system automatically detects when and where the observation was made from the exchangeable image file format (EXIF) information and these data are stored in the system’s data server. Collaboration is undertaken with regional event managers such as teachers and museum curators and field observation events conducted with citizen scientists using our web-based system (Osawa et al. 2013). The purpose of the events depends largely on the collaborators and varies according to whether the event is scientifically or educationally orientated. Consequently, this project has covered several themes and has yielded data on various taxonomic groups in many regions scattered across Japan (Osawa et al. 2013). Although these records contain information on a wide range of organisms in many regions, when combined with other biodiversity records, they can help to clarify regional flora or fauna and nationwide distribution ranges.

In this data paper, the collection of both plant and insect observation records are reported based on photos taken by many citizen scientists. All records have the location and date/time, which were extracted from the EXIF information in the header of the photo files. Although observation records, collected by citizen scientists, may have quality problems, such as misidentification (Osawa 2015), photographic data can be useful for avoiding such problems. The compiliation of digital data records according to Darwin Core, which is a standard format for biodiversity data, have also been reported.

## Project description

### Title

NIAES mobile photo project

### Study area description

This project does not have specific themes, such as target species. Instead, the themes are chosen by regional project managers, who have their own targets, motivations and purposes. Thus, the project was designed to include a variety of approaches, have different goals and involve many scientific disciplines (Sasaki et al. 2016, Kobori et al. 2015). Increasing participation in citizen science projects is essential (Silvertown 2009) and devising strategies on how to attract participants and deciding which themes will enhance participation are critical challenges (Kobori et al. 2015).

### Design description

The platform has been provided (described in the Sampling methods section) for an internet-based photo-collection system for regional managers. Regional managers have been assisted in arranging data collection events, but the data collection itself is handled by the regional managers. For example, the manager could be a natural history museum curator who is interested in organising nature walks that incorporate photography, or an environmental non-profit organisation that is conducting a survey of alien invasive species around a locality. Each regional manager arranges the data collection method, recruits participants and posts photos to this system via e-mail. Those e-mails are then automatically handled by the system and are stored in the system’s data server.

## Sampling methods

### Sampling description

**Figure 1.**
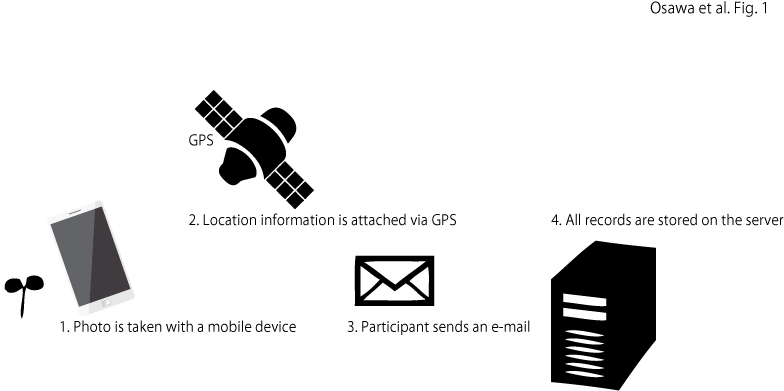
Steps in the data-collection system

A web-based system has been developed to collect photos taken by citizen scientists. The system is a customised version of the commercial mobile photo system developed by Fujitsu FIP, Co. (http://www.fujitsu.com/jp/group/fip/solutions/business-and-technology-solutions/sustainability-solution/management/biodiversity/; accessed 10 August 2017). Customised system could set a several subsystem which independent in each. Each regional manager can prepare that system according to the purpose of the collection event and manage it. In the subsystem, the regional manager can manage the photos which are collected for their event only. In the main system which is managed by the authors, all photos are collected by all subsystems. The data collection procedure is simple: a participant takes a photo of the observation target and sends an e-mail with the photo that has geographic information embedded by GPS (Fig. 1). The timing and location of all the photos sent to the system are automatically extracted and stored in the data server (Fig. 1). The records and the photos are available to be viewed by participants in these project websites from 2011 to 2016 (Note, however, that they are currently closed.) with the map of the web GIS.

The rationale for each event are discussed with the regional manager beforehand and an event-specific subsystem for projects is established. An event-specific address to which the participants send their e-mails is designated. Event-specific websites which relate to each subsystem are opened. For some observation events, citizen scientists are asked to identify the organisms that they have observed and put the names of the organisms in their e-mails. Regional managers are helped to check all records posted and re-examine the species names classified by the citizen scientists on the basis of the photos attached. Records that could not be identified to the species level from a photo are removed.

## Geographic coverage

### Description

The collection sites of the data provided here are distributed across Japan, from Hokkaido to the southern islands. One foreign record is also included.

### Coordinates

 and Latitude; and Longitude.

## Taxonomic coverage

### Description

With regard to taxonomy and systematics, all species were identified by the regional managers and authors according to the morphotypes of objects. If sufficient information could not be obtained for proper species identification, i.e. the species which is difficult to identify, that record was not included in this data paper. As a result, 843 records of 154 species have been provided. Thus, these species were ordinary which can identify easily based on photo.

## Temporal coverage

### Notes

The system was launched in 2011. Data will be released from 2011 to 2016.

## Usage rights

### Use license

Open Data Commons Attribution License

## Data resources

### Data package title

A crowdsourcing approach to collecting photo-based insect and plant observation records

### Resource link

https://www.gbif.org/dataset/c671d43f-7fb9-4b5a-a964-630bfbf47dd2#geographicCoverages

### Number of data sets

1

### Data set 1.

#### Data set name

dwca-niaes_mobile_photopj1-v1.3.zip

#### Data format

Darwin Core Archive

#### Number of columns

16

#### Download URL

http://www.gbif.jp/ipt/archive.do?r=niaes_mobile_photopj1

#### Description

The data set is available from GBIF network through Japan node of GBIF(JBIF).

**Data set 1.** 

Column label	Column description
occurrenceID	An identifier for the Occurrence (photo).
institutionID	An identifier for the institution having custody of the information.
collectionCode	Identifying the collection from which the record was derived. In this data set, project name is provided.
basisOfRecord	The specific nature of the data record. In this data set, all records were "Observation".
scientificName	The scientific name without authors and year.
taxonRank	Lowest taxonomic rank of the record. In this data set, all records were species.
kingdom	Kingdom name.
phylum	Phylum name. Some of species were not ranked.
class	Class name. Some of species were not ranked.
order	Order name.
family	Family name.
eventDate	The date on which a photo was taken.
decimalLatitude	Approximate point latitude of the field site in decimal degrees.
decimalLongitude	Approximate point longitude of the field site in decimal degrees.
verbatimCoordinateSystem	The spatial coordinate system on that data set.
geodeticDatum	Geodetic datum

## References

[B3787938] Dickinson Janis L., Zuckerberg Benjamin, Bonter David N. (2010). Citizen science as an ecological research tool: challenges and benefits. Annual Review of Ecology, Evolution, and Systematics.

[B3787948] Dickinson Janis L, Shirk Jennifer, Bonter David, Bonney Rick, Crain Rhiannon L, Martin Jason, Phillips Tina, Purcell Karen (2012). The current state of citizen science as a tool for ecological research and public engagement. Frontiers in Ecology and the Environment.

[B3787962] Kobori Hiromi, Dickinson Janis L., Washitani Izumi, Sakurai Ryo, Amano Tatsuya, Komatsu Naoya, Kitamura Wataru, Takagawa Shinichi, Koyama Kazuo, Ogawara Takao, Miller-Rushing A. J. (2015). Citizen science: a new approach to advance ecology, education, and conservation. Ecological Research.

[B3787979] Miyazaki Yusuke, Murase Atsunobu, Sahara Ryosuke, Angulo Arturo, Senou Hiroshi (2016). Adding fish images taken in other countries to the biodiversity database of a Japanese public museum, with report of range extension of* Labrisomus jenkinsi* from the Pacific coast of Costa Rica. Ecological Research.

[B3812579] Miyazaki Yusuke, Murase Atsunobu, Shiina Masato, Naoe Kenichi, Nakashiro Ryosuke, Honda Junichi, Yamaide Junichiro, Senou Hiroshi (2014). Biological monitoring by citizens using Web-based photographic databases of fishes. Biodiversity and Conservation.

[B3787990] Osawa Takeshi (2013). Monitoring records of plant species in the Hakone region of Fuji-Hakone-Izu National Park, Japan, 2001–2010. Ecological Research.

[B3788000] Osawa Takeshi (2015). Importance of farmland in urbanized areas as a landscape component for barn swallows (*Hirundo
rustica*) nesting on concrete buildings. Environmental Management.

[B3788032] Osawa T., Yamanaka T., Nakatani Y. (2013). Establishing a canonical procedure for collecting biodiversity information from citizen scientists using mobile phones. Japanese Journal of Conservation Ecology.

[B3788042] Sasaki H., Ohnishi W., Osawa T. (2016). Redefining ‘citizen science’ from several perspectives. Japanese Journal of Conservation Ecology.

[B3788010] Silvertown Jonathan (2009). A new dawn for citizen science. Trends in Ecology & Evolution.

[B3788020] Sullivan Brian L., Wood Christopher L., Iliff Marshall J., Bonney Rick E., Fink Daniel, Kelling Steve (2009). eBird: A citizen-based bird observation network in the biological sciences. Biological Conservation.

